# Modelling Methane Production and Sulfate Reduction in Anaerobic Granular Sludge Reactor with Ethanol as Electron Donor

**DOI:** 10.1038/srep35312

**Published:** 2016-10-12

**Authors:** Jing Sun, Xiaohu Dai, Qilin Wang, Yuting Pan, Bing-Jie Ni

**Affiliations:** 1 State Key Laboratory of Pollution Control and Resources Reuse, National Engineering Research Center for Urban Pollution Control, College of Environmental Science and Engineering, Tongji University, Shanghai 200092, PR China; 2 Advanced Water Management Centre, The University of Queensland, St. Lucia, Brisbane, QLD 4072, Australia; 3 Department of Environmental Science and Engineering, School of Architecture and Environment, Sichuan University, Chengdu, Sichuan 610065, China

## Abstract

In this work, a mathematical model based on growth kinetics of microorganisms and substrates transportation through biofilms was developed to describe methane production and sulfate reduction with ethanol being a key electron donor. The model was calibrated and validated using experimental data from two case studies conducted in granule-based Upflow Anaerobic Sludge Blanket reactors. The results suggest that the developed model could satisfactorily describe methane and sulfide productions as well as ethanol and sulfate removals in both systems. The modeling results reveal a stratified distribution of methanogenic archaea, sulfate-reducing bacteria and fermentative bacteria in the anaerobic granular sludge and the relative abundances of these microorganisms vary with substrate concentrations. It also indicates sulfate-reducing bacteria can successfully outcompete fermentative bacteria for ethanol utilization when COD/SO_4_^2−^ ratio reaches 0.5. Model simulation suggests that an optimal granule diameter for the maximum methane production efficiency can be achieved while the sulfate reduction efficiency is not significantly affected by variation in granule size. It also indicates that the methane production and sulfate reduction can be affected by ethanol and sulfate loading rates, and the microbial community development stage in the reactor, which provided comprehensive insights into the system for its practical operation.

Anaerobic granular sludge reactors, many in the form of Upflow Anaerobic Sludge Blanket (UASB) reactors, are most widely used anaerobic biological reactors treating industrial wastewater high in organic compounds or inorganic compounds such as sulfate^1^,^2^. The high density of microbial communities in anaerobic granular sludge enables the high removal efficiency, low operational cost and great energy generation potential in the system^3^,^4^. In anaerobic granular sludge reactors, organic matter in the wastewater is firstly hydrolyzed and fermented into acetate and hydrogen by the fermentative bacteria (FB), which are then used for methane production through the metabolism of methanogenic archaea (MA)^5^. In the presence of sulfate, sulfate-reducing bacteria (SRB) can inhabit the anaerobic granular sludge using hydrogen and acetate for sulfate reduction and this results in competition with MA^6^. This competition has been studied intensively in the past, with SRB believed to out-compete MA under sulfate-rich conditions^7^,^8^,^9^. In fact, apart from hydrogen and acetate, SRB could also utilize other fermentable compounds like fatty acids, alcohols (i.e. ethanol), sugars and amino acids for sulfate reduction^6^,^10^,^11^, which would result in the competition between SRB and FB. The competition between SRB and FB was expected to affect the electron donor available for methanogenesis, and might consequently affect the competition between SRB and MA. It could also pose impact on the sulfate reduction pathways and sulfate reduction efficiency in the system, due to the existence of different electron donors. Therefore, the degradation of these fermentable organic compounds by different microorganisms needs to be comprehensively studied when assessing methane production and sulfate reduction in anaerobic granular sludge reactors.

Ethanol is such an important fermentable compound that can be readily consumed by both SRB and FB. It has been widely detected in the industrial wastewater discharged from chemical units, brewery factories, or pharmaceutical factories with substantially high concentrations and is regarded as a preferable carbon source for methane production^12^,^13^. In recent years, experimental studies have been carried out to investigate the methane generation capability through fermenting ethanol in granule-based UASB reactors^14^,^15^. Hu *et al*.^15^ found that the methane production decreased with increasing sulfate loading in the reactor, as ethanol could be partially oxidized by SRB to acetate. In addition, anaerobic ethanol degradation is also an important process in anaerobic granular sludge reactors treating high-sulfate wastewater. Though the cost of ethanol needs to be considered during the practical operation, it is still a common industrial practice to add ethanol into anaerobic granular sludge systems as a supplement of carbon source for sulfate reduction, mostly because ethanol can be readily used by SRB and its price is low^13^,^16^. The effectiveness of ethanol utilization by SRB would directly affect the sulfate removal efficiency in the system. Thus, thoroughly understanding the degradation of ethanol in the anaerobic granular sludge reactor and its effect on methane production and sulfate reduction are of great importance for industrial application.

Mathematically modelling is a powerful tool to investigate different microbial processes and their interactions in a bioreactor. With a proper model, methane production and sulfate reduction with different substrates could be predicted without massive experimental inputs. Also, the competition between different microorganisms involved could be investigated. So far, models have been established to mainly simulate methane production and sulfate reduction using acetate and hydrogen as substrates, with some including sulfate reduction by fatty acids^17^,^18^,^19^,^20^. However, though ethanol plays an important role in methane production and sulfate reduction in anaerobic granular sludge systems as aforementioned, the ethanol-involved microbial processes with regard to methane production and sulfate reduction have not been specifically modelled. Especially, the competition on ethanol by FB and SRB has not been elucidated by previous models^17^,^18^. In addition, in the anaerobic granular sludge system, the differences in substrates penetration also need to be considered in the model, as it could significantly affect the distribution pattern of different microorganisms in the granular sludge.

Therefore, this study aims to develop a mathematic model to describe the methane production and sulfate reduction in anaerobic granular sludge reactors using ethanol as a key electron donor and carbon source. The validity and applicability of the model were tested by comparing model simulations with experimental data from two granule-based UASB reactor studies, which were operated for 375 days and 180 days, respectively (hereinafter referred as to “Study Case I” and “Study Case II”). The UASB reactors in these two studies both had a working volume of 6 L, which were operated independently under different conditions. Model-based analysis was also carried out to investigate microbial communities in the granular sludge and factors affecting performance of the anaerobic granular sludge reactor, including granular size, sulfate loading rates and ethanol loading rates, which provides useful information for optimizing methane production or sulfate removal processes in the practical application.

## Results

### Model evaluation with experimental data of Study Case I

**Figure 1:**
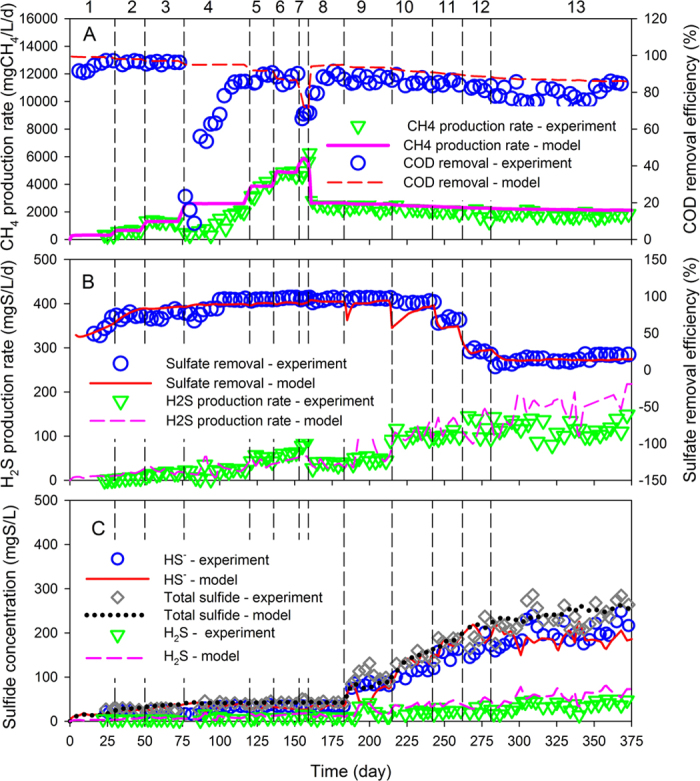
Experimental measured and model simulated reactor performance in Study Case I. (**A**) Methane production rate and COD removal efficiency; (**B**) H2S production rate and sulfate removal efficiency; and (**C**) hydrogen sulfide (H2S), bisulfide ion (HS^−^) and total sulfide (H_2_S + HS^−^) profiles in the effluent. The vertical dashed lines separated different experimental phases as described in Section 2.2 and Supplementary Table S5.

The model describing methane production and sulfate reduction using ethanol as a key electron donor was schematically presented in Supplementary Fig. S1, Supplementary Information. The stoichiometry of components and the kinetic expressions of each process involved in the model with the values of model parameters were summarized in Supplementary Tables S1–S4. The developed model was firstly evaluated with experimental data of Study Case I^15^, in which the granular-based UASB reactor was feed with synthetic wastewater containing approximately 1000 mg/L of ethanol and 1000 mg/L of acetate. The organic loading rate (OLR) varied from 1.4 to 36.6 gCOD/L/d and the sulfate concentration changed from 150 mg/L to 6000 mg/L according to the experimental design (See Methods Section). The whole study period was divided into two experiments. The model was firstly calibrated to describe the data in the first experiment (Day 0–159), which was then validated using the data obtained from the second experiment (Day 160–375). The calibrated values of μ_XF_, K_XF,E_, μ_XSRBE_, K_XSRBE,SO4_ giving the optimum model fittings were listed in Supplementary Table S4. The model predictions and experiment measurements of the reactor performance in terms of methane production and sulfate reduction are compared in Fig. 1.

As shown in Fig. 1A, during the first experimental period (Day 0–159), the model predicted methane production rate rose stepwise with the increase of OLR, well predicting the trend obtained by experimental measurements. Except for on day 75–85, the experimental data were not captured by the model, because the reactor performance failure occurred due to a sharp decrease in pH caused by insufficient addition of NaHCO_3_^15^ and this effect was not included in the model. The modelling profiles showed that COD removal efficiency was maintained over 90% when the OLR increased from 1.4 gCOD/L/d to 18.8 gCOD/L/d and decreased slightly with the increase of OLR to 25.2 gCOD/L/d, but plunged to ~65% when OLR reached up to 36.6 gCOD/L/d, suggesting that the maximum COD removal ability has already been achieved with this OLR. The mismatch of the modelling and experimental obtained COD removal in Day 75–85 was also due to the deterioration of the reactor performance caused by pH drops as discussed above. In the second experiment period (Day 160–375), the modelling methane production rate decreased marginally with the increase of sulfate in the influent, while the COD removal ratio maintained at around 80% during the whole period. This indicated that more COD was used for sulfate reduction with the increasing inlet sulfate concentration. The model well predicted experimental trends.

The model predicted and experimental measured H_2_S production and sulfate removal in the reactor were illustrated in Fig. 1B. During Experiment 1 (Day 0–159), the H_2_S production rate and the sulfate removal efficiency increased gradually due to the development of ethanol-utilizing SRB (ESRB) and hydrogen-utilizing SRB (HSRB) in the anaerobic granular sludge (see details in the Discuss Section), and then reached a relatively steady level. During Experiment 2 (Day 160–375), the H_2_S production rate was progressively enhanced with the increasing sulfate concentration in the influent. However, gradually decrease of the sulfate removal efficiency was observed. Figure 1C shows the profiles of the hydrogen sulfide (H_2_S), bisulfide ion (HS^−^) and total sulfide (H_2_S + HS^−^) concentrations in the effluent, which followed the same trend as variation of H_2_S production rate as shown in Fig. 1B. The increase of inlet sulfate concentration substantially raised the bisulfide ion concentration and the total sulfide concentration. The hydrogen sulfide concentration also increased but to a less extent, as the pH of ~7.5 in the reactor favored the dissociation of sulfide into bisulfide ion. The model well described these observations.

The results suggested that the developed model captured the experimental data reasonably well (R^2^ > 0.8). The good agreement between model simulations and measured data indicated that the model could properly describe the relationships among methane production and sulfate reduction during the routine operation in Study Case I.

### Model evaluation with experimental data of Study Case II

**Figure 2:**
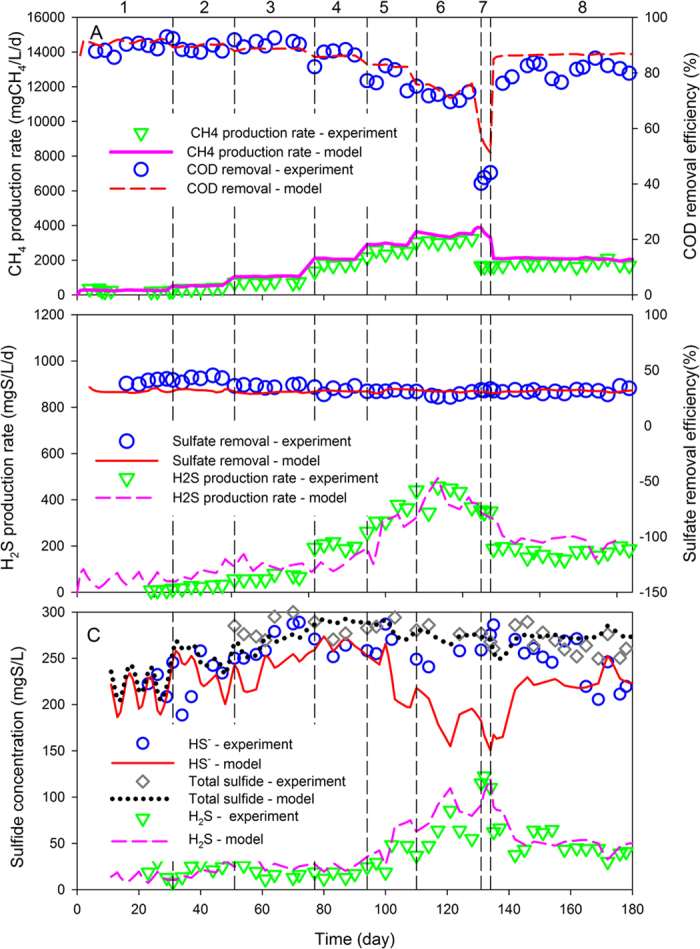
Experimental measured and model simulated reactor performance in Study Case II. (**A**) Methane production rate and COD removal efficiency; (**B**) H2S production rate and sulfate removal efficiency; and (**C**) hydrogen sulfide (H_2_S), bisulfide ion (HS^−^) and total sulfide (H_2_S + HS^−^) profiles in the effluent. The vertical dashed lines indicate different experimental phases as described in Section 2.2 and Supplementary Table S5.

The developed model was further evaluated and validated by the experimental data obtained from Study Case II^14^, using the same parameter values as applied in Study Case I. Compared with Study Case I, Study Case II had different combinations of OLR and sulfate loading rates (SLR). In addition, the influent sulfate concentration (3000 mgSO_4_/L) in the Study Case II was much higher than that in the Study Case I for most of the experimental duration. The COD/SO_4_^2−^ ratio in the influent was maintained at the same level during the whole experimental period in Study Case II, while the overall loading increased step by step and then declined to a steady level. Figure 2 indicated that the model simulation could reasonably reflect the experimental measured reactor performance with regard to methane production and sulfate reduction in Study Case II.

Specifically, the methane production rate increased progressively with the increase of OLR and declined to a constant state with the OLR falling and then staying at a lower level (Fig. 2A). In contrast, the COD removal efficiency decreased gradually with increasing OLR and plummeted when OLR reached 37.5 gCOD/L/d. It was then recovered to around 80% when the OLR went down back to 12 gCOD/L/d. The H_2_S production rate also increased gradually with the increase of SLR, while the sulfate removal efficiency maintained constantly at ~35% during the whole experimental period (Fig. 2B). Figure 2C shows that in the effluent, the total sulfide concentration did not significantly change with OLR variation, while the hydrogen sulfide increased from Day 95–131 and then decreased and maintained at steady level. However, on Day 100–160, the modelled bisulfide ion profile had a decrease whereas the measured data stayed at the same level (Fig. 2C). The differences between the model predictions and experimental data probably were due to the measurement errors in bisulfide ion concentration, as according to the experimental measurement, the balance of reduced sulfur species (Total sulfide concentration = Bisulfide concentration + Hydrogen sulfide concentration) could not be reached.

The agreement between model simulations and all the measured results at different OLR levels was generally good for all fitted variables (R^2^ > 0.8), suggesting this developed model was also able to describe the experimental data from Study Case II, which further supported the validity of the developed model.

## Discussion

### Distribution and evolution of microbial communities

**Figure 3:**
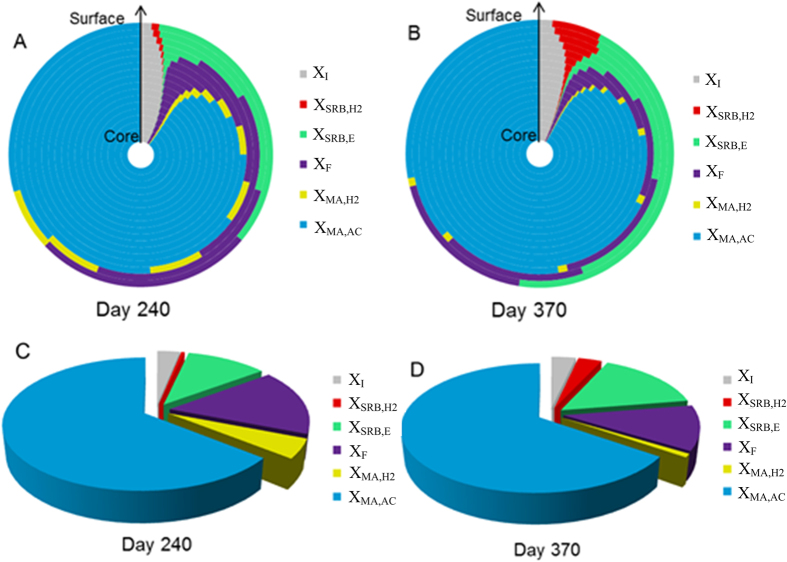
(**A**,**B**) The simulated distribution of key microorganisms from the core to the surface of the anaerobic granular sludge on Day 240 (**A**) and Day 370 (**B**) in Study Case I. The areas of different colors indicate the relative abundances of corresponding microorganisms in each layer from the core of the granule to the surface. (**C**,**D**) The simulated proportion of different microorganisms in the anaerobic granular sludge on Day 240 (**C**) and Day 370 (**D**).

Both model simulations and experimental results suggested that changes of influent ethanol and sulfate concentrations or their loading rates would pose significant impact on methane production and sulfate reduction in the anaerobic granular sludge reactor. This could be related to the evolution of microbial communities in the anaerobic granular sludge under different substrate conditions^21^,^22^,^23^. Since it was difficult to precisely determine the changes of the microbial populations and community structures in the granular sludge, simulation study was carried out for further investigation. The microbial structure of the anaerobic granular sludge on two key stages, i.e., Day 240 and Day 370 in the Study Case I, were simulated and compared in Fig. 3. In general, the acetated-utilizing MA (AMA) had the highest abundance (represented over 60% of the total microbial population) in the anaerobic granular sludge and dominated the core of the granule on both days. In contrast, other microorganisms including FB, hydrogen-utilizing MA (HMA), HSRB and ESRB accounted for about 30% of the total microbial populations and mainly situated in the outer layer of the anaerobic granular sludge. This stratified distribution pattern of microorganisms in the anaerobic granular sludge was consistent with those reported in anaerobic aggregates, biofilms and sediments producing methane and sulfide^11^,^24^,^25^.

From Day 240 to Day 370, the population of ESRB and HSRB increased noticeably with the increase of sulfate concentration in the influent. On the other hand, the abundance of FB and HMA decreased accordingly, most likely due to the substrates for FB and HMA were competitively used by SRB with the increase of sulfate loading. This could be explained by the kinetics advantages of SRB over FB and HMA, with the ESRB having a higher growth rate than the FB, while the HSRB having a both higher growth rate and affinity to hydrogen than HMA, as listed in Supplementary Table S4. The variation in the population of FB and ESRB from Day 240 to Day 370 was consistent with the change of ethanol consumption rates by FB and ESRB as shown in the Supplementary Fig. S2. The ethanol consumption rate by FB decreased gradually while that by ESRB increased significantly, resulting in much higher ethanol consumption rate by ESRB than that by FB on Day 370. This suggested that ESRB could successfully outcompete FB under COD/SO_4_^2−^ of 0.5. The inhibitory effects of H_2_S on HSRB and HMA are not expected to significantly affect the competition between these two microorganisms as HSRB has a higher tolerance to H_2_S than HMA^26^. In addition, the dissolved H_2_S concentration in the experimental UASB reactors was lower than 50 mgS/L, much lower than the reported inhibition constant (Supplementary Table S4). Thus, the dissolved H_2_S would not cause significant inhibitory effect on SRB and MA activities in the studied systems. The model simulation suggested the evolution of microbial communities in the anaerobic granular sludge from Day 240 to Day 370 is in good agreement with the changes in reactor performance (Fig. 1).

The acetate-utilizing SRB (ASRB) were not existed in the system, as suggested by the model simulation (Fig. 3) as well as cloning analysis results^15^. This could be due to the low initial abundance of acetate-utilizing SRB in the inoculated granule and its relatively low growth rate^10^. This is in good agreement with which reported by other researchers. Omil *et al*.^21^ found it took over 200 days before ASRB outcompeting AMA in a UASB reactor when operating in exceed of sulfate. O’Flaherty *et al*.^27^ even observed that AMA can effectively compete ASRB in an anaerobic biofilm reactor after five years operation. As suggested by the model simulation, the high-sulfate conditions (COD/SO_4_^2^ = 0.5) in Study Case I were not long enough for the development of acetate-utilizing SRB in the system. Modeling results revealed the ASRB could grow in the system with prolonged operational time under COD/SO_4_^2−^ of 0.5 (Supplementary Fig. S3), and the sulfate reduction activities were also enhanced accordingly.

### Impact of granule size on methane production and sulfate reduction

**Figure 4:**
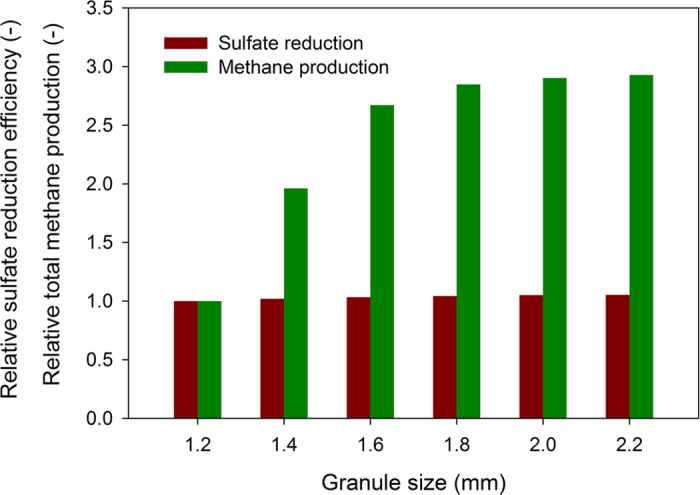
Impact of granular size on sulfate reduction and methane production in the anaerobic granular sludge reactor.

Granule size was considered as one of the key parameters affecting the performance in anaerobic granular sludge reactor^28^,^29^. Therefore, methane production and sulfate reduction in the anaerobic granular sludge reactor with the average granule diameters in a range from 1.2 mm to 2.2 mm were simulated and compared. In the simulation, the influent concentrations of the substrates were set at 1000 mgCOD/L for acetate, 2000 mgCOD/L for ethanol and 1500 mgSO_4_/L for sulfate. The HRT was set at 6 h. As shown in Fig. 4, the sulfate reduction efficiency was not significantly affected by variation in granule size, while the total methane production declined obviously when the granular diameter decreased from 1.8 mm to 1.2 mm. The different impacts of the granular size on sulfate reduction and methane production could be related to the stratified distribution pattern of SRB and MA in the granular sludge (Fig. 3A,B), which was mainly caused by the kinetic difference of SRB and MA in substrates (acetate and hydrogen) utilization and the penetration limitation of substrates in the granule. Since SRB has kinetic advantages over MA on their common substrates^7^,^8^,^9^, the SRB zone would be determined by sulfate input into the granule rather than the granule size. In the outer layer of the granule where the substrates for SRB could be penetrated in, SRB can easily outcompete MA and become dominating. Accordingly, the decrease of granule size would not affect the SRB zone as the sulfate input did not change at different granule size conditions. In contrast, most MA would situate inside the granular sludge where SRB were hardly developed since sulfate was depleted by the sulfate reducing activity in the outer layer. When the diameter of the granular sludge decreased, the activities and the abundance of SRB did not change, leading to the fact that SRB would occupy more relative space fraction in the small size granule than that in large size granule. For example, our modelling results show that when the diameter of the granular sludge decreased from 1.8 mm to 0.8 mm, the activities and the abundance of SRB showed same level. As a result, less space would be available for the growth of MA with the decrease of the granular diameter and the methane production decreased accordingly. However, increase of granular diameter from 1.8 mm to 2.4 mm did not markedly enhance the methane production any further (Fig. 4). This was probably due to the fact that the penetration limitation of substrate for methanogensis has been reached with the tested COD loading. Because of the lack of substrate, cells inside the granules might decay and leak out inert cellular products. Therefore, granules with a larger diameter might contain a higher inert fraction in the center but a similar methane production capacity^29^,^30^.

Hence, there should be an optimal granule diameter for the maximum methane production efficiency. For a larger granule diameter, a higher diffusion-resistant level had to be overcome. Because of substrate limitations, cells inside the granules might decay and leak out inert cellular products. Thus, granules with a larger diameter contained a higher inert fraction and thereby a decreased volumetric activity. For this reason, the point on the plateaus of the methane production in Fig. 4 could be recognized as the critical point, to which an optimal granule diameter corresponds. The simulation reveals that the optimum diameter for methane production in our reactor under the simulated operating conditions should be around 1.8 mm. It should be noted that the optimum diameter of 1.8 mm here was not always suitable to all anaerobic granular system. It was found that the average granule diameter might fluctuate in time in real experimental systems. The simulation data in this work strongly suggests that it is correlated with methane production. Thus, the optimum diameter for methane production of the anaerobic granule was related to the operation conditions of the reactor. In addition, with the proceeding of granulation, the biomass was progressively stratified with the anaerobic granules settled in the lower part of the reaction zone in UASB. When granules were formed increasingly in the reaction zone, a dense sludge bed and a thin sludge blanket were formed with a clear interface between them in UASB, which resulted in a decreasing of granule size with the increasing of the height of the reactor.

### Impacts of ethanol and sulfate loading rates

**Figure 5:**
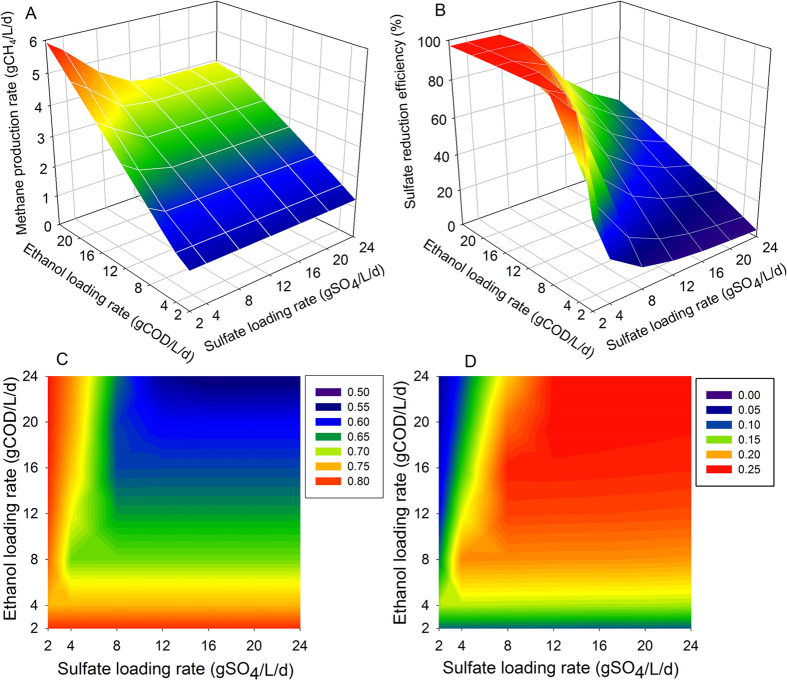
Model simulated methane production rate (**A**) and sulfate reduction efficiency (**B**) and the proportion of COD used for methane production (**C**) and sulfate reduction (**D**) with different combination of ethanol loading rate (ELR) and sulfate loading rate (SLR) at the early stage without ASRB.

**Figure 6:**
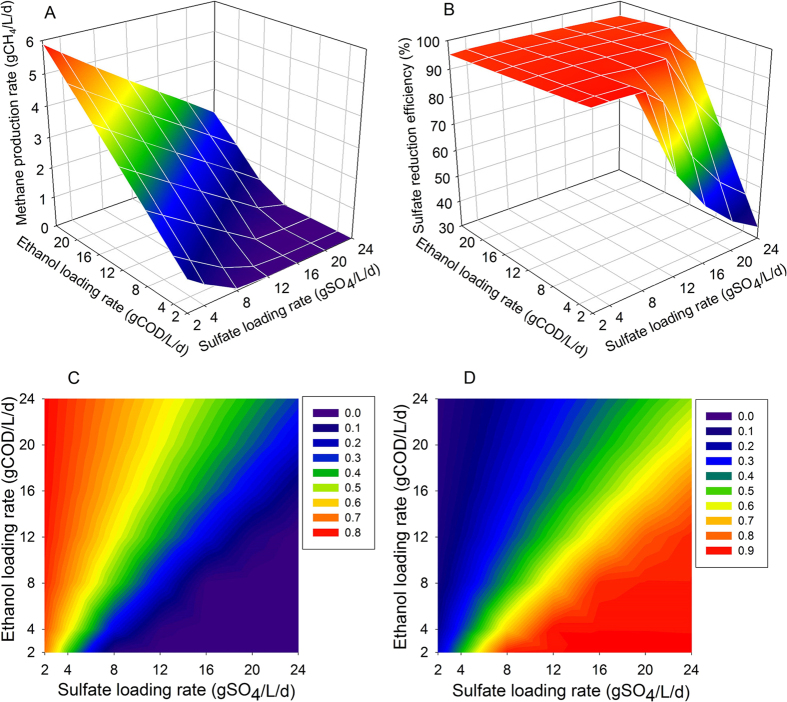
Model simulated methane production rate (**A**) and sulfate reduction efficiency (**B**) and the proportion of COD used for methane production (**C**) and sulfate reduction (**D**) with different combination of ethanol loading rate (ELR) and sulfate loading rate (SLR) at the steady state.

To further investigate the impact of substrates loading on the system, the methane production and sulfate reduction with different combination of ethanol loading rates (ELR, ranged from 2 to 24 gCOD/L/d) and sulfate loading rates (SLR, ranged from 2 to 24 gSO_4_/L/d) were simulated and compared. The tested ELR and SLR cover a wide range in order to offer different options for real application. As previously discussed in the Section of “Distribution and evolution of microbial communities”, at the early stage, ASRB might not develop in the anaerobic granular sludge due to its low growth rate and low abundance. However, when the system reached the steady state after it was operated for a long period from its starting up, the ASRB might out-compete AMA and contribute significantly on sulfate reduction. Therefore, the effect of ELR and SLR on the reactor performance at both early microbial community development stage (no ASRB has been developed) and steady state (possibly with ASRB depending on substrates loading) were studied and illustrated in Figs 5 and 6, respectively.

As shown in Fig. 5A, at the early microbial community development stage, the minimum methane production rate was about 1.3 gCH_4_/L/d at an ELR of 2 gCOD/L/d and it rose to 4–6 gCH_4_/L/d at an ELR of 24 gCOD/L/d, depending on the SLR in the system. When the ELR was higher than 8 gCOD/L/d, the increase of SLR resulted in a decline in methane production rate firstly before it maintaining at a steady level. This was probably because that, with the increase of SLR, more COD was used by SRB for sulfate reduction, and thereby, less COD was available for methane production. However, when the maximum sulfate reduction activities were reached, further increase in SLR would not affect the methane production from the system^31^,^32^. This was also explained why the variation in SLR did not significantly affect the methane production rate when the ELR was lower than 8 gCOD/L/d.

Figure 5B presented the sulfate reduction efficiency at the early microbial community development stage with different combinations of ELR and SLR. The results suggested sulfate reduction efficiency increased with the increase of ELR to SLR ratio. To achieve total sulfate reduction, the ELR (represented by COD equivalent) to SLR ratio of 3 was required, which was much higher than the theoretical ratio of COD loading to sulfate loading for sulfate reduction^15^. This was due to that the COD in the form of acetate produced by ethanol degradation could not be used for sulfidogenesis during the early stage owing to lack of ASRB. Consequently, extra COD supplied by ethanol or hydrogen was required for total sulfate reduction.

The percentage of total COD used for methane production and sulfate reduction were calculated to quantify the competition between SRB and MA, as shown in Fig. 5C,D, respectively. At the early microbial community development stage, the fraction of COD used for sulfate reduction varied from 0.05 to 0.25 with different ELR and SLR combinations, while the proportions of COD used for methane production were between 0.55 and 0.80. This indicated that, at the early stage, when the ASRB have not developed, MA can effectively compete with SRB for COD to produce methane even under sulfate-rich conditions.

The methane production rate and sulfate reduction efficiency at the steady state were distinctive from what found at the early stage, as shown in Fig. 6. The methane production rate was positively related to the ELR in the system but negatively related to SLR (Fig. 6A). The maximum methane production rate was closed to 6 gCH_4_/L/d observed with highest ELR (24 gCOD/L/d) and lowest SLR (2 gSO_4_/L/d) in the system. When ELR to SLR ratio was lower than 0.75, the methane production ceased and all the COD in the system was supposed to be utilized for sulfate reduction. This hypothesis could be supported by the changes in sulfate reduction ratio with ELR and SLR in the system (Fig. 6B). Total sulfate reduction was achieved when the ELR to SLR ratio was higher than 0.75 and the rest of COD component could then be used for methanogenesis. The percentages of COD used for methane production and sulfate reduction at the steady state were calculated and presented in Fig. 6C and Fig. 6D, respectively. With the decrease of the ELR to SLR ratio, the percentage of COD for sulfate reduction increased while for methane production declined accordingly. This indicated that SRB could out-compete MA in the sulfate-rich environment when steady state of the anaerobic granular sludge reactor has been reached, which was also consistent with previous studies regarding with competition between SRB and MA in sulfate-rich environment 6,17,21,32.

### Practical implications

In this study, we developed a model to describe methane production and sulfate reduction in the anaerobic granular sludge system with ethanol as a key electron donor and carbon source. As such system has been widely applied to treat wastewater containing both ethanol (either contained or externally added) and sulfate, discharged from chemical units, brewery factories, pharmaceutical factories or other industrial parks^12^,^13^, the developed model could be served as a useful tool for providing guidance during practical operation. The modeling results suggested that the methane production and sulfate reduction in anaerobic granular system would be affected by the size of the anaerobic granular sludge, the ELR to SLR ratio, and microbial community development stage in the system. Therefore, it is necessary to select suitable operational parameters according to different operation conditions or aims.

For organic degradation and methane production purpose, an optimal granular diameter needs to be selected during the reactor operation, as the methane production rate would enhanced by increasing granular size but within a certain value. As shown in Fig. 4, the point on the plateaus of the methane production could be recognized as the critical point, to which an optimal granule diameter corresponded. The simulation revealed that the optimum diameter for methane production in the reactor under the simulated conditions should be between 1.6 and 1.8 mm. The granule larger than the optimal size would contain a higher inert fraction and thereby a decreased volumetric activity^29^,^33^. Methane production would also be affected by the SLR of the system. The modeling results at different microbial community development stages in the reactor suggested the short-term increase of SLR in the reactor would not significantly affect the methane production rate in the system. The reactor treating wastewater high in ethanol and low in sulfate could have a capacity to resist the sulfate loading shock, as MA could effectively compete with SRB for COD at early stage without developing ASRB^14^,^15^. However, after long-term exposal to high sulfate, ASRB are likely to outcompete AMA and the methane production in the system might be suppressed depending on the ELR to SLR ratio^6^,^17^,^21^,^32^.

For the sulfate removal purpose, maintaining a smaller granular size in the anaerobic granular sludge reactor would be recommended, which could be achieved by increasing upflow rate of the influent^34^. As discussed in Section 4.2, the decrease of granular diameter would not significantly affect the sulfate removal ratio but would largely decrease the methane production due to lack of space for the growth of MA. Thus, a small granular size would enhance the proportion of COD utilized for sulfate reduction and less COD would be required for sulfate reduction. The cost for additional COD dosing could consequently be reduced. In addition, the modeling results suggested that ASRB would contribute significantly on sulfate reduction in the system and largely increased the sulfate reduction ratio when steady state of reactor has been reached. However, it might take extremely long period to reach the steady state due to the low growth rate of the ASRB^21^,^27^. Hence, in order to ensure high sulfate reduction efficiency, high abundance of ASRB in the inoculated sludge/granules would be preferred. This kind of inoculum could be obtained from sludge/granules in sulfate-rich environment, while sludge/granules in UASB reactor treating high organic strength wastewater without sulfate may not be suitable^19^,^35^.

## Methods

### Modelling ethanol bioconversion

Under anaerobic conditions with the presence of sulfate, ethanol could either be partially degraded by SRB to produce sulfide and acetate or be fermented into acetate and hydrogen through the metabolism of FB (Equations 1 and 2)^36^,^37^. The produced acetate and hydrogen could then be used for methanogensis or sulfidogenesis, while the sulfide formed in the system might pose inhibitory effects on SRB and MA. The proposed model synthesized all these relevant reactions as summarized in Supplementary Fig. S1.


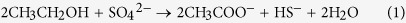



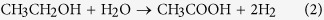


As listed in Supplementary Table S1, the model mainly described the relationships among seven particulate groups, i.e. FB (X_F_), SRB grown on ethanol, acetate and hydrogen, (X_SRB,E_, X_SRB,AC_ and X_SRB,H_), MA grown on acetate and hydrogen (X_MA,AC_ and X_MA,H_) and inert residual biomass (X_I_) and six soluble compound, namely, ethanol (S_E_), acetate (S_AC_), hydrogen (S_H_), sulfate (S_SO4_), sulfide (S_H2S_) and methane (S_CH4_). The unit was gS/m^3^ for all sulfur species while concentrations of other compounds were quantified based their chemical oxygen demand (COD) equivalent, i.e. gCOD/m^3^. Four groups of biological processes were considered in the model, including fermentation, sulfidogenesis, methanogenesis and decay of the biomass. The stoichiometric matrix of the model was summarized in Supplementary Table S2.

The rates of growth of the microorganisms were modelled using Monod-type kinetics while the microorganism decay was simulated through first order kinetics as suggested in the Anaerobic Digestion Model No. 1 (ADM1)^38^. The kinetic expressions of the reactions included in the model were listed in Supplementary Table S3. The inhibitory effects of sulfide on SRB and MA were also modelled through non-competitive inhibition models^39^. In this study, we used the same inhibition constant for all SRB and also the same inhibition constant for the two MA to simplify our model, as the dissolved H_2_S concentration was not high enough to cause significant inhibitory effect in the system. When the H_2_S reached a significant high level, the different inhibitory effect on the three SRB or two MA should be included in the model by applying distinctive inhibition constants to improve the predictions of the model.

### Anaerobic granular sludge reactor model

To simulate the anaerobic granular sludge reactor, a one dimensional granule-based model was utilized according to Ni *et al*.^33^. The granule was assumed in a spherical shape and an average granule diameter was applied based on the experimental measurements. The number of granules was calculated according to Gonzalez-Gil *et al*.^40^ and the biomass in the granules was fixed without migration. The diffusive transportation of soluble components into the granule was described by Fick’s law (refer to Supplementary Equation S1). Discretization in time of the partial-differential equation was applied to describe the reaction-diffusion kinetics in a spherical granular particle^33^. One-dimensional conservation law was formulated as a balance between the mass conserved and utilized in the model^41^. The UASB reactor was modeled using a well-established approach in literature, with subsequent plug flow reactor to the continuously stirred tank reactors in series^42^. An additional mixed compartment was implemented to represent the gas phase, with diffusive links to simulate the gas-liquid mass transfer processes of CH_4_, and H_2_S. The fractions of hydrogen sulfide (H_2_S), bisulfide ion (HS^−^) in the liquid phase were modelled through dissociation equilibrium of sulfide calculated based on acidity coefficient (pKa) and the pH of the water. The model was implemented in the modified AQUASIM 2.1 d software package^43^.

### Experimental data for model evaluation

Long-term experimental data from two study cases with different operational conditions, aiming to investigate the effect of ethanol and sulfate in the influent on methane production and sulfate reduction, were used to test the predictive abilities of the developed model.

#### Study Case I

The study was conducted in a UASB reactor with a working volume of 6 L under 35 °C. The reactor was inoculated with 3 L^15^ mesophilic granular sludge harvested from a full-scale UASB reactor treating food manufacturing wastewater. Synthetic wastewater containing approximately 1000 mg/L of ethanol and 1000 mg/L of acetate mimicking effluent from an organic chemical manufacturing was fed into the reactor. The sulfate concentration was adjusted by adding sodium sulfate (Na_2_SO_4_). The pH was controlled in the reactor by adding NaHCO_3_ at 1500 mg/L on Day 0–80 and at 3000 mg/L on Day 80–375. Two sets of experiments were carried out in this study. The organic loading rate, the sulfate concentration in the influent and the HRT of each experimental period were listed in Supplementary Table S5. In the first experiment (Day 0–159), the OLR increased stepwise from 1.4 to 36.6 gCOD/L/d with the sulfate concentration being maintained at 150 mg SO_4_/L. In the second experiment (Day 160–375), the OLR was kept at 12 gCOD/L/d and the sulfate concentration increased gradually from 150 mg SO_4_/L to 6000 mg SO_4_/L. Liquid samples were taken for the measurement of COD and sulfur species. The compositions of methane and sulfide in the biogas were also monitored.

#### Study Case II

The study was carried in a UASB reactor having a reacting zone with height of 0.8 m and volume of 6 L^14^. Three liter of seeding granular sludge from a full-scale UASB reactor treating was inoculated to the reactor. The average diameter and sedimentation velocity of the granules were about 1.8 mm and 96.5 m/h, respectively. The feeding wastewater contained about 1000 mg/L acetate and 1000 mg/L ethanol. A NaHCO_3_ dosage of 3000 mg/L was added to adjust pH in the system. Compared with Study Case I, Study Case II had a different combination of OLR, influent sulfate concentration and HRT, as also listed in Supplementary Table S5. Specifically, the HRT of this reactor was decreased gradually from 48 to 2 h from Day 0–135 and then kept at 6 h afterwards till Day 180. Accordingly, the OLR increased from 1.4 to 37.8 gCOD/L/d and then stayed at 12.3 gCOD/L/d. The sulfate concentration was kept at 3000 mg SO_4_/L during the whole experiment period. The methane and gaseous sulfide production from the reactor as well as COD and sulfur species in the water phase were measured.

### Model calibration and validation

The developed model includes 32 stoichiometric and kinetic parameters as summarized in Supplementary Table S4. Most model parameter values were well established in previous studies and thus adapted from literature, as presented in Supplementary Table S4. However, limited information is available in literature for the parameters related to ethanol fermentation by FB and ethanol degradation by SRB. Therefore, four key parameters of anaerobic ethanol degradation by FB and SRB were calibrated in the model, including maximum growth rate of X_F_ (μ_XF_), half saturation value of X_F_ for ethanol (K_XF,E_), maximum growth rate of X_SRB,E_ (μ_XSBE_) and half saturation value of X_SRB,E_ for sulfate (K_XSRBE,SO4_). The experimental data obtained in the first experiment (Day 0–159) from Study Case I were used for parameter estimation, which was conducted by minimizing the sum of squares of the deviations between the measured data and the model predictions. The experimental data obtained in the second experiment (Day 160–375) from Study Case I were then used for model and parameters validation. To further verify and validate the model applicability, the model was also applied to evaluate the experimental data from Study Case II with the same parameter values obtained from Study Case I.

## Additional Information

**How to cite this article**: Sun, J. *et al*. Modelling Methane Production and Sulfate Reduction in Anaerobic Granular Sludge Reactor with Ethanol as Electron Donor. *Sci. Rep.* **6**, 35312; doi: 10.1038/srep35312 (2016).

## Supplementary Material

Supplementary Information

## References

[b1] Abbasi T. & Abbasi S. A. Formation and impact of granules in fostering clean energy production and wastewater treatment in upflow anaerobic sludge blanket (UASB) reactors. Renew. Sust. Energ. Rev. 16, 1696–1708 (2012).

[b2] Lu X. . Operation performance and granule characterization of upflow anaerobic sludge blanket (UASB) reactor treating wastewater with starch as the sole carbon source. Bioresource Technol. 180, 264–273 (2015). 10.1016/j.biortech.2015.01.010 25617619

[b3] Chan Y. J., Chong M. F., Law C. L. & Hassell D. G. A review on anaerobic–aerobic treatment of industrial and municipal wastewater. Chem. Eng. J. 155, 1–18 (2009).

[b4] Liu Y., Zhang Y. & Ni B.-J. Zero valent iron simultaneously enhances methane production and sulfate reduction in anaerobic granular sludge reactors. Water Res. 75, 292–300 (2015). 25867207 10.1016/j.watres.2015.02.056

[b5] Liu Y. & Tay J.-H. State of the art of biogranulation technology for wastewater treatment. Biotechnol. Adv. 22, 533–563 (2004). 15262316 10.1016/j.biotechadv.2004.05.001

[b6] Muyzer G. & Stams A. J. M. The ecology and biotechnology of sulphate-reducing bacteria. Nat. Rev. Micro. 6, 441–454 (2008). 10.1038/nrmicro1892 18461075

[b7] Schönheit P., Kristjansson J. K. & Thauer R. K. Kinetic mechanism for the ability of sulfate reducers to out-compete methanogens for acetate. Arch. Microbiol. 132, 285–288 (1982).

[b8] Miller T. L., Wolin M. J., Demacario E. C. & Macario A. J. L. Isolation of Methanobrevibacter-Smithii from Human Feces. Appl. Environ. Microb. 43, 227–232 (1982). 10.1128/aem.43.1.227-232.1982 PMC241804 6798932

[b9] Raskin L., Rittmann B. & Stahl D. Competition and Coexistence of Sulfate-Reducing and Methanogenic Populations in Anaerobic Biofilms. Appl. Environ. Microbiol. 62, 3847–3857 (1996). 16535428 10.1128/aem.62.10.3847-3857.1996 PMC1388966

[b10] Barton L. L. & Fauque G. D. Biochemistry, Physiology and Biotechnology of Sulfate-Reducing Bacteria. Advances in Applied Microbiology, Vol 68, 41–98 (2009). 19426853 10.1016/S0065-2164(09)01202-7

[b11] Sun J., Hu S., Sharma K. R., Ni B.-J. & Yuan Z. Stratified Microbial Structure and Activity in Sulfide- and Methane-Producing Anaerobic Sewer Biofilms. Appl. Environ. Microb. 80, 7042–7052 (2014). 10.1128/AEM.02146-14 PMC4249016 25192994

[b12] Nagpal S., Chuichulcherm S., Livingston A. & Peeva L. Ethanol utilization by sulfate-reducing bacteria: An experimental and modeling study. Biotechnol. Bioeng. 70, 533–543 (2000). 11042550

[b13] Kaksonen A., Franzmann P. & Puhakka J. Performance and Ethanol Oxidation Kinetics of a Sulfate-Reducing Fluidized-Bed Reactor Treating Acidic Metal-Containing Wastewater. Biodegradation 14, 207–217 (2003). 12889611 10.1023/a:1024262607099

[b14] Jing Z. . UASB performance and electron competition between methane-producing archaea and sulfate-reducing bacteria in treating sulfate-rich wastewater containing ethanol and acetate. Bioresource Technol. 137, 349–357 (2013). 10.1016/j.biortech.2013.03.137 23597763

[b15] Hu Y. . Effect of influent COD/SO_4_^2‒^ ratios on UASB treatment of a synthetic sulfate-containing wastewater. Chemosphere 130, 24–33 (2015). 25747303 10.1016/j.chemosphere.2015.02.019

[b16] Kalyuzhnyi S. V., De Leon Fragoso C. & Rodriguez Martinez J. Biological sulfate reduction in a UASB reactor fed with ethanol as the electron donor. Microbiology 66, 562–567 (1997).

[b17] Kalyuzhnyi S. V. & Fedorovich V. V. Mathematical modelling of competition between sulphate reduction and methanogenesis in anaerobic reactors. Bioresource Technol. 65, 227–242 (1998).

[b18] Fedorovich V., Lens P. & Kalyuzhnyi S. Extension of Anaerobic Digestion Model No. 1 with processes of sulfate reduction. Appl. Biochem. Biotech. 109, 33–45 (2003). 10.1385/abab:109:1-3:33 12794282

[b19] Knobel A. N. & Lewis A. E. A mathematical model of a high sulphate wastewater anaerobic treatment system. Water Res. 36, 257–265 (2002). 11766803 10.1016/s0043-1354(01)00209-3

[b20] Guisasola A., Sharma K. R., Keller J. & Yuan Z. Q. Development of a model for assessing methane formation in rising main sewers. Water Res. 43, 2874–2884 (2009). 19423146 10.1016/j.watres.2009.03.040

[b21] Omil F., Lens P., Visser A., Hulshoff Pol L. W. & Lettinga G. Long-term competition between sulfate reducing and methanogenic bacteria in UASB reactors treating volatile fatty acids. Biotechnol. Bioeng. 57, 676–685 (1998). 10099247

[b22] Ito T., Okabe S., Satoh H. & Watanabe Y. Successional Development of Sulfate-Reducing Bacterial Populations and Their Activities in a Wastewater Biofilm Growing under Microaerophilic Conditions. Appl. Environ. Microbiol. 68, 1392–1402 (2002). 11872492 10.1128/AEM.68.3.1392-1402.2002 PMC123773

[b23] Kobayashi T., Yan F., Takahashi S. & Li Y.-Y. Effect of starch addition on the biological conversion and microbial community in a methanol-fed UASB reactor during long-term continuous operation. Bioresource Technol. 102, 7713–7719 (2011). 10.1016/j.biortech.2011.05.084 21700450

[b24] Koizumi Y., Takii S., Nishino M. & Nakajima T. Vertical distributions of sulfate-reducing bacteria and methane-producing archaea quantified by oligonucleotide probe hybridization in the profundal sediment of a mesotrophic lake. Fems Microbiol. Ecol. 44, 101–108 (2003). 19719655 10.1016/S0168-6496(02)00463-4

[b25] Santegoeds C. M. . Distribution of Sulfate-Reducing and Methanogenic Bacteria in Anaerobic Aggregates Determined by Microsensor and Molecular Analyses. Appl. Environ. Microb. 65, 4618–4629 (1999). 10.1128/aem.65.10.4618-4629.1999 PMC91616 10508098

[b26] Yamaguchi T., Harada H., Hisano T., Yamazaki S. & Tseng I. C. Process behavior of UASB reactor treating a wastewater containing high strength sulfate. Water Res. 33, 3182–3190 (1999).

[b27] O’Flaherty V., Lens P., Leahy B. & Colleran E. Long-term competition between sulphate-reducing and methane-producing bacteria during full-scale anaerobic treatment of citric acid production wastewater. Water Res. 32, 815–825 (1998).

[b28] Bhunia P. & Ghangrekar M. M. Required minimum granule size in UASB reactor and characteristics variation with size. Bioresource Technol. 98, 994–999 (2007). 10.1016/j.biortech.2006.04.019 16781145

[b29] Ahn Y., Song Y. J., Lee Y. J. & Park S. Physicochemical Characterization of UASB Sludge with Different Size Distributions. Environ. Technol. 23, 889–897 (2002). 12211449 10.1080/09593332308618356

[b30] Liu Y., Xu H.-L., Yang S.-F. & Tay J.-H. Mechanisms and models for anaerobic granulation in upflow anaerobic sludge blanket reactor. Water Res. 37, 661–673 (2003). 12688701 10.1016/s0043-1354(02)00351-2

[b31] Moosa S., Nemati M. & Harrison S. T. L. A kinetic study on anaerobic reduction of sulphate, Part I: Effect of sulphate concentration. Chem. Eng. Sci. 57, 2773–2780 (2002).

[b32] Chou H.-H., Huang J.-S., Chen W.-G. & Ohara R. Competitive reaction kinetics of sulfate-reducing bacteria and methanogenic bacteria in anaerobic filters. Bioresource Technol. 99, 8061–8067 (2008). 10.1016/j.biortech.2008.03.044 18448334

[b33] Ni B.-J. . Modeling a granule-based anaerobic ammonium oxidizing (ANAMMOX) process. Biotechnol. Bioeng. 103, 490–499 (2009). 19280667 10.1002/bit.22279

[b34] Tiwari M. K., Guha S., Harendranath C. S. & Tripathi S. Influence of extrinsic factors on granulation in UASB reactor. Appl. Microbiol. Biot. 71, 145–154 (2006). 10.1007/s00253-006-0397-3 16607526

[b35] Liu B. . Effects of ethanol/SO_4_^2−^ ratio and pH on mesophilic sulfate reduction in UASB reactors. Afr. J. Microbiol. Res. 4, 2215–2222 (2010).

[b36] Liamleam W. & Annachhatre A. P. Electron donors for biological sulfate reduction. Biotechnol. Adv. 25, 452–463 (2007). 17572039 10.1016/j.biotechadv.2007.05.002

[b37] Bryant M. P., Wolin E. A., Wolin M. J. & Wolfe R. S. Methanobacillus omelianskii, a symbiotic association of two species of bacteria. Archiv für Mikrobiologie 59, 20–31 (1967). 5602458 10.1007/BF00406313

[b38] Batstone D. J. . Anaerobic Digestion Model No. 1 (ADM1). (ed. Batstone D. J. .) (IWA Publishing, 2002).

[b39] Kaksonen A. H., Franzmann P. D. & Puhakka J. A. Effects of hydraulic retention time and sulfide toxicity on ethanol and acetate oxidation in sulfate-reducing metal-precipitating fluidized-bed reactor. Biotechnol. Bioeng. 86, 332–343 (2004). 15083513 10.1002/bit.20061

[b40] Gonzalez-Gil G., Seghezzo L., Lettinga G. & Kleerebezem R. Kinetics and mass-transfer phenomena in anaerobic granular sludge. Biotechnol. Bioeng. 73, 125–134 (2001). 11255160 10.1002/bit.1044

[b41] Wanner O. & Reichert P. Mathematical modeling of mixed-culture biofilms. Biotechnol. Bioeng. 49, 172–184 (1996). 18623567 10.1002/(SICI)1097-0290(19960120)49:2<172::AID-BIT6>3.0.CO;2-N

[b42] Ren T.-T., Mu Y., Ni B.-J. & Yu H.-Q. Hydrodynamics of upflow anaerobic sludge blanket reactors. Aiche J. 55, 516–528 (2009).

[b43] Batstone D. J., Keller J. & Blackall L. L. The influence of substrate kinetics on the microbial community structure in granular anaerobic biomass. Water Res. 38, 1390–1404 (2004). 15016516 10.1016/j.watres.2003.12.003

