# Supplementary material for: Modelling Methane Production and Sulfate Reduction in Anaerobic Granular Sludge Reactor with Ethanol as Electron Donor
Source: Sci Rep. 2016 Oct 12;6:35312. doi: 10.1038/srep35312 (PMC5059677; doi:10.1038/srep35312)
Supplement: Supplementary Information [file srep35312-s1.pdf]

Supplementary Information

Modelling Methane Production and Sulfate Reduction in Anaerobic Granular Sludge Reactor with Ethanol as Electron Donor

Jing Sun[1], Xiaohu Dai[1], Qilin Wang[2], Yuting Pan[3], Bing-Jie Ni[1,*]

[1]State Key Laboratory of Pollution Control and Resources Reuse, National Engineering Research Center for Urban Pollution Control, College of Environmental Science and Engineering, Tongji University, Shanghai 200092, PR China

[2]Advanced Water Management Centre, The University of Queensland, St. Lucia, Brisbane, QLD 4072, Australia

[3]Department of Environmental Science and Engineering, School of Architecture and Environment, Sichuan University, Chengdu, Sichuan 610065, China

***Corresponding author:**

Bing-Jie Ni, P: +86 21 65986849; F: +86 21 65983602; E-mail: bjni@tongji.edu.cn

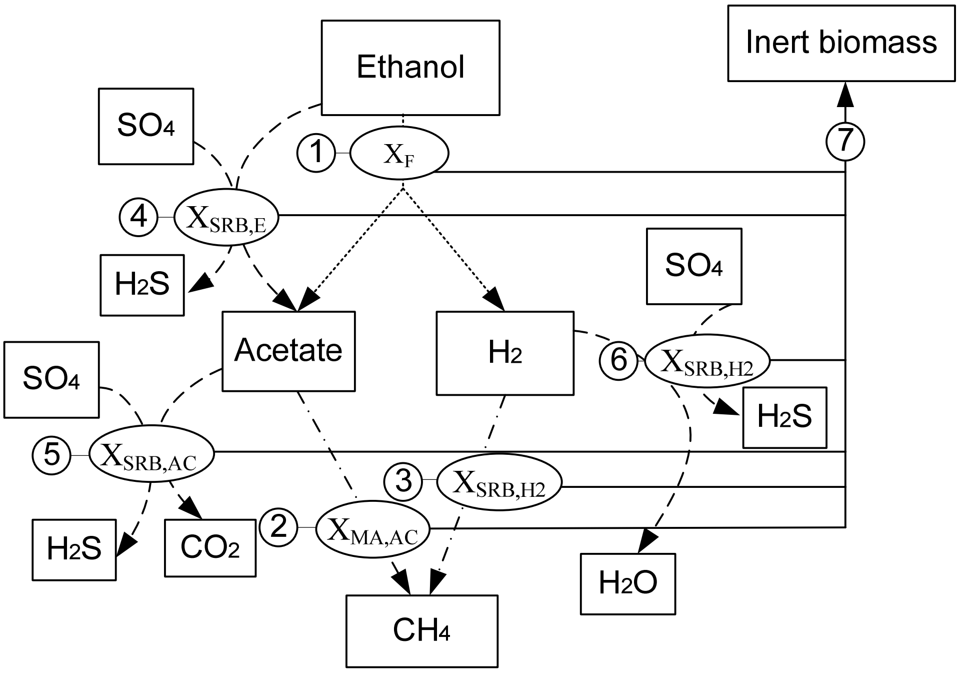

Supplementary Figure S1. Schematic of biochemical processes included in the developed model: ①Ethanol fermentation ② Methanogenesis from acetate; ③ Methanogenesis from hydrogen; ④ Sulfidogenesis from ethanol; ⑤Sulfidogenesis from acetate; ⑥Sulfidogenesis from hydrogen; and ⑦Decay of the biomass.

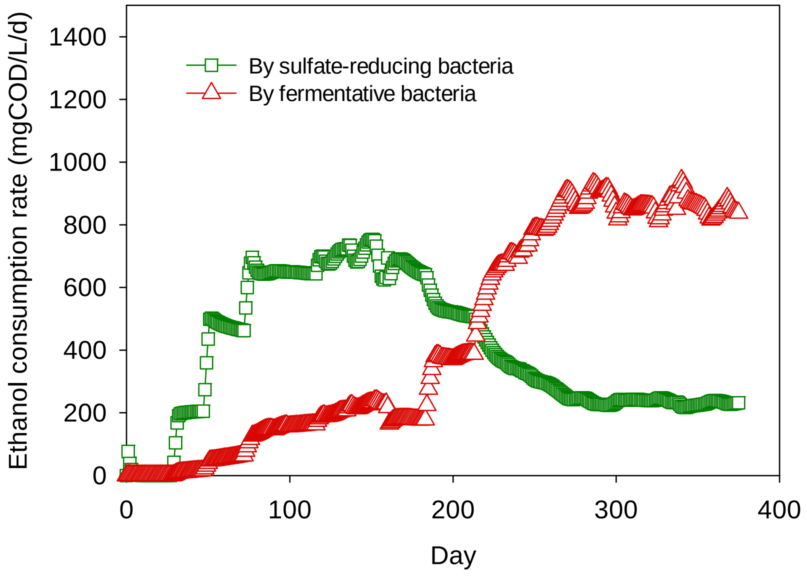

Supplementary Figure S2. The ethanol consumption rates by fermentative bacteria (FB) and ethanol-utilizing sulfate reducing bacteria (ESRB) in Study Case I.

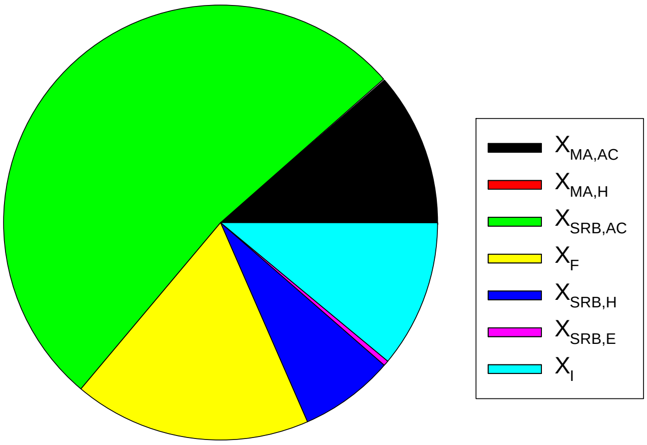

Supplementary Figure S3. The simulated proportion of different microorganisms in the anaerobic granular sludge on Day 500.

Supplementary Table S1. The definition and units of model components.

Variable	Description	Unit
S_E	Ethanol	$gCOD/m^3$
S_{AC}	Acetate	$gCOD/m^3$
S_{H2}	Hydrogen	$gCOD/m^3$
S_{CH4}	Methane	$gCOD/m^3$
S_{SO4}	Sulfate	gS/m^3
S_{H2S}	Sulfide	gS/m^3
X_F	Fermentative bacteria	$gCOD/m^3$
$X_{SRB,E}$	SRB grown on ethanol	$gCOD/m^3$
$X_{SRB,AC}$	SRB grown on acetate	$gCOD/m^3$
$X_{SRB,H2}$	SRB grown on H_2	$gCOD/m^3$
$X_{MA,AC}$	MA grown on acetate	$gCOD/m^3$
$X_{MA,H2}$	MA grown on H_2	$gCOD/m^3$
X_I	Inert particular COD	$gCOD/m^3$

Supplementary Table S2. Stoichiometry of the developed ethanol bioconversion model.

No	Process	S_E	S_{AC}	S_{H2}	S_{SO4}	S_{H2S}	S_{CH4}	X_I	X_F	$X_{SRB,E}$	$X_{SRB,H}$	$X_{SRB,AC}$	$X_{MA,AC}$	$X_{MA,H}$
1	Ethanol fermentation	$-\frac{1}{Y_{XH}}$	$\frac{2}{3}(\frac{1-Y_{XH}}{Y_{XH}})$	$\frac{1}{3}(\frac{1-Y_{XH}}{Y_{XH}})$					1					
2	Methanogenesis using acetate		$-\frac{1}{Y_{XMAAC}}$				$\frac{1-Y_{XMAAC}}{Y_{XMAAC}}$						1	
3	Methanogenesis using hydrogen			$-\frac{1}{Y_{XMAH}}$			$\frac{1-Y_{XMAH}}{Y_{XMAH}}$							1
4	Sulfate reduction using ethanol	$-\frac{1}{Y_{XSRBE}}$	$\frac{2}{3}(\frac{1-Y_{XSRBE}}{Y_{XSRBE}})$		$-\frac{1}{6}(\frac{1-Y_{XSRBE}}{Y_{XSRBE}})$	$\frac{1}{6}(\frac{1-Y_{XSRBE}}{Y_{XSRBE}})$				1				
5	Sulfate reduction using acetate		$-\frac{1}{Y_{XSRBAC}}$		$-\frac{1}{2}(\frac{1-Y_{XSRBAC}}{Y_{XSRBAC}})$	$\frac{1}{2}(\frac{1-Y_{XSRBAC}}{Y_{XSRBAC}})$						1		
6	Sulfate reduction using hydrogen			$-\frac{1}{Y_{XSRBH}}$	$-\frac{1}{2}(\frac{1-Y_{XSRBH}}{Y_{XSRBH}})$	$\frac{1}{2}(\frac{1-Y_{XSRBH}}{Y_{XSRBH}})$					1			
7	Decay of X_F							f	-1					
8	Decay of X_{SRBE}							f		-1				
9	Decay of X_{SRBH}							f			-1			
10	Decay of X_{SRBAC}							f				-1		
11	Decay of X_{MAAC}							f					-1	
12	Decay of X_{MAH}							f						-1

Supplementary Table S3. Kinetic expressions of the developed model.

No.	Processes	Kinetic expressions
1	Ethanol fermentation	$m_{XH} \cdot \frac{S_E}{K_{\mathrm{E}} + S_E} \cdot X_H$
2	Sulfate reduction using ethanol	$m_{XSRB,E} \cdot \frac{S_E}{K_{XSRB,E} + S_E} \cdot \frac{S_{SO4}}{K_{XSRB,E,SO4} + S_{SO4}} \cdot \frac{K_{I,SRB}}{K_{I,SRB} + S_{H2S}} \cdot X_{SRB,E}$
3	Sulfate reduction using hydrogen	$m_{XSRB,H} \cdot \frac{S_H}{K_{XSRB,H} + S_H} \cdot \frac{S_{SO4}}{K_{XSRB,H,SO4} + S_{SO4}} \cdot \frac{K_{I,SRB,H2S}}{K_{I,SRB,H2S} + S_{H2S}} \cdot X_{SRB,H}$
4	Sulfate reduction using acetate	$m_{XSRB,AC} \cdot \frac{S_{AC}}{K_{XSRB,AC} + S_{AC}} \cdot \frac{S_{SO4}}{K_{XSRB,AC,SO4} + S_{SO4}} \cdot \frac{K_{I,SRB,H2S}}{K_{I,SRB,H2S} + S_{H2S}} \cdot X_{SRB,AC}$
5	Methanogenesis using acetate	$m_{XMA,AC} \cdot \frac{S_{AC}}{K_{XMA,AC} + S_{AC}} \cdot \frac{K_{I,MA,H2S}}{K_{I,MA} + S_{H2S}} \cdot X_{MA,AC}$
6	Methanogenesis using h2	$m_{XMA,AC} \cdot \frac{S_H}{K_{XMA,H} + S_H} \cdot \frac{K_{I,MA,H2S}}{K_{I,MA,H2S} + S_{H2S}} \cdot X_{MA,H}$
7	Decay of X_F	$kdec_{XH} \cdot X_H$
8	Decay of X_{SRBE}	$kdec_{XSRB,E} \cdot X_{SRB,E}$
9	Decay of X_{SRBH}	$kdec_{XSRB,H} \cdot X_{SRB,H}$
10	Decay of X_{SRBAC}	$kdec_{XSRB,AC} \cdot X_{SRB,AC}$
11	Decay of X_{MAAC}	$kdec_{XMA,AC} \cdot X_{MA,AC}$
12	Decay of X_{MAH}	$kdec_{XMA,H} \cdot X_{MA,H}$

Supplementary Table S4. Kinetic and stoichiometric parameters of the developed model.

Parameter	Description	Unit	Value	Reference
m_{XE}	Maximum growth rate of X_E	d^{-1}	0.5	calibrated
$K_{XE,E}$	Half saturation value of X_E for S_E	$gCOD/m^3$	4.3	calibrated
$m_{XMA,AC}$	Maximum growth rate of $X_{M,AC}$	d^{-1}	0.21	1
$K_{XMA,AC}$	Half saturation value of $X_{MA,AC}$ for S_{AC}	$gCOD/m^3$	76	2 ,3
$m_{XMA,H2}$	Maximum growth rate of $X_{MA,H2}$	d^{-1}	1.2	1
$K_{XMA,H2}$	Half saturation value of $X_{MA,H2}$ for S_{H2}	$gCOD/m^3$	0.04	3
$m_{XSRB,AC}$	Maximum growth rate of $X_{SRB,AC}$	d^{-1}	0.55	1
$K_{XSRB,AC}$	Half saturation value of $X_{SRB,AC}$ for S_{AC}	$gCOD/m^3$	6	1
$K_{XSRB,AC,SO4}$	Half saturation value of $X_{SRB,AC}$ for S_{SO4}	gS/m^3	3.2	4
$m_{XSRB,H2}$	Maximum growth rate of $X_{SRB,H2}$	d^{-1}	1.37	3
$K_{XSRB,H2}$	Half saturation value of $X_{SRB,H2}$ for S_{H2}	$gCOD/m^3$	0.03	3
$K_{XSRB,H2,SO4}$	Half saturation value of $X_{SRB,H2}$ for S_{SO4}	gS/m^3	3.3	4
$m_{XSRB,E}$	Maximum growth rate of $X_{SRB,H2}$	d^{-1}	1.41	calibrated
$K_{XSRB,E}$	Half saturation value of $X_{SRB,H2}$ for S_{H2}	$gCOD/m^3$	8.6	5
$K_{XSRB,E,SO4}$	Half saturation value of $X_{SRB,H2}$ for S_{SO4}	gS/m^3	1.5	calibrated
$kdec_{XH,AC}$	First order decay rate of $X_{H,AC}$	d^{-1}	0.02	2
$kdec_{XH,PROP}$	First order decay rate of $X_{H,PROP}$	d^{-1}	0.02	2
$kdec_{XMA,AC}$	First order decay rate of $X_{MA,AC}$	d^{-1}	0.02	2
$kdec_{XMA,H2}$	First order decay rate of $X_{MA,H2}$	d^{-1}	0.02	2
$kdec_{XSRB,F}$	First order decay rate of $X_{SRB,F}$	d^{-1}	0.02	2
$kdec_{XSRB,AC}$	First order decay rate of $X_{SRB,AC}$	d^{-1}	0.02	2
$kdec_{XSRB,H2}$	First order decay rate of $X_{SRB,H2}$	d^{-1}	0.02	2
Y_{XE}	Yield of X_E	gCOD/gCOD	0.06	6
$Y_{XMA,AC}$	Yield of $X_{MA,AC}$	gCOD/gCOD	0.05	2
$Y_{XMA,H2}$	Yield of $X_{MA,H2}$	gCOD/gCOD	0.06	2
$Y_{XSRB,AC}$	Yield of $X_{SRB,AC}$	gCOD/gCOD	5.68×10^{-2}	1
$Y_{XSRB,H2}$	Yield of $X_{SRB,H2}$	gCOD/gCOD	7.53×10^{-2}	1
$Y_{XSRB,E}$	Yield of $X_{SRB,PROP}$	gCOD/gCOD	0.06	7
$K_{I,H2S,MA}$	Inhibition constant of MA activity by H_2S	gS/m^3	160	8
$K_{I,H2S,SRB}$	Inhibition constant of SRB activity by H_2S	gS/m^3	270	8
f	Fraction of X_I generated due to biomass decay	gCOD/gCOD	0.08	9
D_{H2S}	Diffusive coefficient of H_2S in water	m^2/d	1.38×10^{-4}	10
D_{SO4}	Diffusive coefficient of sulfate in water	m^2/d	9.16×10^{-5}	10

D_{H2}	Diffusive coefficient of hydrogen in water	m^2/d	3.89×10^{-4}	10
D_{AC}	Diffusive coefficient of acetate in water	m^2/d	1.05×10^{-4}	10
D_E	Diffusive coefficient of ethanol in water	m^2/d	9.38×10^{-5}	11
D_{CH4}	Diffusive coefficient of methane in water	m^2/d	1.29×10^{-4}	10
k^0H	Henry’s law constant of hydrogen sulfide	$mol/m^3/Pa$	1.0×10^{-3}	12

Supplementary Table S5. Experimental conditions in Study Case I and Study Case II.

Study Case I					Study Case II				
Phase	Day	HRT (h)	OLR ($gCODL^{-1}d^{-1}$)	SO_4^{2-} (mg/L)	Phase	Day	HRT (h)	OLR ($gCODL^{-1}d^{-1}$)	SO_4^{2-} (mg/L)
1	0-29	48	1.4	150	1	0-29	48	1.4	3000
2	30-49	24	2.9	150	2	30-47	24	2.9	3000
3	50-75	12	6.0	150	3	48-72	12	6.0	3000
4	76-119	6	11.9	150	4	73-90	6	12.0	3000
5	120-135	4	18.8	150	5	91-108	4	18.0	3000
6	136-152	3	25.2	150	6	109-128	3	24.7	3000
7	153-159	2	36.6	150	7	129-134	2	37.8	3000
8	160-182	6	12.0	150	8	135-179	6	12.3	3000
9	183-215	6	12.0	300					
10	216-242	6	12.0	600					
11	243-262	6	12.0	1000					
12	263-281	6	12.0	3000					
13	282-375	6	12.0	6000					

Supplementary Equation S1: diffusion equation of soluble substrate into biofilms:

$$\frac{\partial S}{\partial t} = D_f \frac{\partial^2 S}{\partial x^2}$$

Where:

S is the concentration of the soluble substrate (kg/m^3);

t is time (d) ;

D_f is the diffusion coefficient of the substrate in the biofilm (m^2/d), which are set at 0.8-fold of the values in the water[13];

x is the depth into the biofilm (m).

References

1 Kalyuzhnyi, S., Fedorovich, V., Lens, P., Po, L. H. & Lettinga, G. Mathematical modelling as a tool to study population dynamics between sulfate reducing and methanogenic bacteria. *Biodegradation* **9**, 187-199, (1998).

2 Batstone, D. J. *et al.* The IWA Anaerobic Digestion Model No. 1 (ADM1). *Water Sci. Technol.* **45**, 75-83 (2002).

3 Harada, H., Uemura, S. & Momonoi, K. Interaction between sulfate-reducing bacteria and methane-producing bacteria in UASB reactors fed with low strength wastes containing different levels of sulfate. *Water Res.* **28**, 355-367, (1994).

4 Fedorovich, V., Lens, P. & Kalyuzhnyi, S. Extension of Anaerobic Digestion Model No. 1 with processes of sulfate reduction. *Appl. Biochem. Biotech.* **109**, 33-45, (2003).

5 Kaksonen, A., Franzmann, P. & Puhakka, J. Performance and Ethanol Oxidation Kinetics of a Sulfate-Reducing Fluidized-Bed Reactor Treating Acidic Metal-Containing Wastewater. *Biodegradation* **14**, 207-217, (2003).

6 Cord-Ruwisch, R., Lovley, D. R. & Schink, B. Growth of Geobacter sulfurreducens with Acetate in Syntrophic Cooperation with Hydrogen-Oxidizing Anaerobic Partners. *Appl. Environ. Microb.* **64**, 2232-2236 (1998).

7 Nagpal, S., Chuichulcherm, S., Livingston, A. & Peeva, L. Ethanol utilization by sulfate-reducing bacteria: An experimental and modeling study. *Biotechnol. Bioeng.* **70**, 533-543, (2000).

8 Yamaguchi, T., Harada, H., Hisano, T., Yamazaki, S. & Tseng, I. C. Process behavior of UASB reactor treating a wastewater containing high strength sulfate. *Water Res.* **33**, 3182-3190, (1999).

9 Ni, B.-J. *et al.* Modeling a granule-based anaerobic ammonium oxidizing (ANAMMOX) process. *Biotechnol. Bioeng.* **103**, 490-499, (2009).

10 Stewart, P. S. Diffusion in Biofilms. *J. Bacteriol.* **185**, 1485-1491, (2003).

11 Westrin, B. A. Measurement of the diffusion coefficient of ethanol in agarose gel beads: A reproducibility study. *Biotechnol. Tech.* **4**, 409-414, (1990).

12 Sander, R. Compilation of Henry's law constants (version 4.0) for water as solvent. *Atmos. Chem. Phys.* **15**, 4399-4981, (2015).

13 Terada, A., Lackner, S., Tsuneda, S. & Smets, B. F. Redox‐stratification controlled biofilm (ReSCoBi) for completely autotrophic nitrogen removal: The effect of co‐versus counter‐diffusion on reactor performance. *Biotechnol. Bioeng.* **97**, 40-51 (2007).
